# Wages or Legitimacy? A Qualitative Analysis of Home Care Worker Perspectives in Choosing Work Settings

**DOI:** 10.1177/00469580241248094

**Published:** 2024-06-06

**Authors:** Christine Kelly, Lisette Dansereau, Jennifer C. H. Sebring, Yeonjung Lee, Allison Williams

**Affiliations:** 1 University of Manitoba, Winnipeg, MB, Canada; 2 Chung-Ang University, Seoul, South Korea; 3 University of Calgary, Calgary, AB, Canada; 4 McMaster University, Hamilton, ON, Canada

**Keywords:** Canada, home care agencies, home care services, home health aides, health workforce

## Abstract

Directly-Funded (DF) home care allows users to organize and purchase their own care services and is expanding globally. Little is known about the career pathways of home care workers. Our study asks, what experiences and factors do home care workers consider when choosing a work setting? And, specifically, what influences their decisions to work directly for their clients? Framed with Cranford’s (2020) flexibility-security matrix for analyzing home care dynamics, we remotely interviewed 20 home care workers in two Canadian provinces. Three team members conducted axial coding and thematic analysis using Dedoose software. We identified personal and material factors at the intimate and labor market level that workers weigh when choosing whether to work for an agency or directly for a client. At the intimate level, workers value the flexibility, autonomy, and respect facilitated in care relations when working directly for a client. At the labor market level, agencies provide better job security and the benefit of supervisory support but lower wages. Additionally, as care work often serves as a stepping stone for immigration and citizenship agency positions are considered a more “legitimate” option than working directly for a client. Our study shows that workers directly employed by their clients enjoy more flexibility but lack security, whereas agency employed workers risk immediate reductions in working conditions in exchange for limited improvements in safety and supervision and, like other frontline care work, DF home care represents a key career pathway for immigrants with previous experience in health and social care settings.

**What do we already know about this topic?**
• There is increased demand for home care workers to support growing populations of older and disabled people to age at home.
• The home care workforce is characterized by high turnover and poor working conditions, with limited research specifically on workers in directly-funded or “cash-for-care” policy contexts.
**What does this study add to existing knowledge?**
• Care workers weigh trade-offs in flexibility and security when choosing work settings.
• Working in residential facilities provide the greatest stability and pay for workers, while there are wide variations and wage disparities in home care.
• Home care, particularly working directly for a client, provides greater autonomy and flexibility for workers.
**What are the key implications for public health interventions, practice or policy?**
• Care worker wages should be standardized across institutional, home care and other settings to support health system transformation toward more home and community-based services.
• Greater recognition of international training and experience is needed.

## Introduction

The demand for frontline, paid care workers is increasing in parallel with growing populations of older and disabled people who need help in daily life.^1^ This labor force is often unregulated and associated with a variety of job titles including: direct care workers, personal support workers, personal care aides, home health aides, and others. In this context, care workers support older adults and disabled people with the activities of daily living in settings across the continuum of care, and provide up to 80% of direct care in Canadian homes and long-term care facilities.^2^ The demand for more care workers to work in home care programs can be situated in national and international policy and health contexts that support a shift away from residential long-term care.^3,4^

Understanding the experiences of care workers is essential to support the increasing demand for home and community care. This workforce is often described as “unstable,” characterized by high turnover, long vacancy periods, and poor working conditions.^5,6^ The majority of care workers are women and are overrepresented by immigrants and/or people of colour.^7^ Additionally a “strikingly” high number of care workers indicate a disability status, with higher prevalence of disability among home care workers in particular.^8^ Strengthening the home care workforce is a priority in health human resource planning and in aims to support equity in population health outcomes, including the health of care workers themselves.

This study examines one of the most precarious employment arrangements for care workers; those working under directly-funded (DF) home care programs.^9^ DF home care allows users to organize and purchase their care services and is expanding globally. For care workers, it often means the client or family *is* the employer, although some programs allow a third-party agency to mediate the relationship. While client and family satisfaction with DF home care programs is well-established,^10,11^ there are concerns about the risks and benefits to care workers who choose this path.

Our study asks, what experiences and factors do home care workers consider when choosing a work setting? And, specifically, what influences their decisions to work directly for their clients? We find workers weigh and balance personal and material trade-offs at the intimate and labor market level when choosing whether to work for an agency in contrast to working directly for a client.

## Relevant Literature

There is a growing international body of research on workforce and human resourcing issues in home care, although little that specifically focuses on DF home care. Below, we summarize key findings related to career trajectories, and employment settings and satisfaction.

### Career Trajectories

Career trajectories are made up of sequences of events and transitions (eg, getting a new job or changing industries) where individuals forge their path in the context of institutional and social constraints.^12^ Trajectories are influenced by individual and demographic factors such as gender, immigration status and family origins,^13^ education and social networks,^14^ and the labor market context.^15^ There are widespread reports of difficulties recruiting care workers, yet one study established a “high willingness to consider working as a paid home care worker” among the general population in New York state.^16^ However, the career prospects are limited and “‘upward’ mobility does not necessarily mean achieving a living wage or exiting poverty.” ^17(p83),18^

Care work may be considered an entry-level job and promoted to recent immigrants as a first step in gaining local work experience. Covington-Ward found direct care jobs are “easily obtained” by African immigrants in the United States, but are “not really a choice” given the urgent need for income and work.^19^ Home care workers are more likely to have “difficulties changing jobs due to having a disability, and lower self-rated health” than direct care workers in institutional health care settings; these factors may affect career advancement, retention, work transitions, and turnover.^5,8(p676),20^ While care work may be “easy” to get into, there is an intensification in skill requirements due to increasing levels of need,^21^ putting more demands on workers. There are also a number of safety–related hazards to working in this sector, including a high prevalence of musculoskeletal injury, high levels of emotional demands and negative mental health impacts.^20,22,23^ In brief, it may be easy to get a job in the home care sector, but it can be challenging work with little prospect of advancement.

### Employment Settings and Satisfaction

Employee satisfaction is a key component supporting workforce recruitment and retention in any field. As compared to providing care in a facility, workers prefer working in the home care sector, reporting better perceptions of support, safety, and engagement.^24,25^ Perhaps key to higher satisfaction working in home care, numerous studies document that workers value the autonomy they experience in home care work.^26-28^ Despite reporting higher job satisfaction and increased autonomy, home care workers receive lower pay and fewer benefits compared to those working in facilities.^29^ Further, a survey of care workers in Ontario suggests that providing training specific to the more complex tasks increasingly expected of care workers increases their overall training satisfaction—one of many factors in job satisfaction.^30^ Retention issues in the home care workforce may thus be best addressed through material improvements in pay and benefits.^26^

There is limited evidence that links increased levels of worker satisfaction to specific types of home care employment arrangements, whether working through direct employment with a client, for a private agency, or for public services. A 2019 report out of the United Kingdom found that direct employment “was much more rewarding and satisfying than working for a care agency.”^31(p11)^ Another study found workers perceived their employment conditions as significantly better in the public sector as compared to the private sector, with little difference whether the private employer was for-profit or nonprofit.^32^ This finding resonates with growing literature questioning the role of private home care providers and reinforces the long-standing importance of material working conditions for job satisfaction.^24,33^

Our study helps respond to the need for further research considering the personal trajectories and choices of care workers, and how individual pathways may relate to where they end up working. While our study takes place in Canada, directly-funded home care exists globally, and our findings add to the growing body of international literature on directly-funded home care and care worker experiences more broadly.

## Methodology

### Research Design and Theoretical Framework

Our study involved semi-structured interviews with care workers employed through DF home care and subsequent thematic analysis. We followed O’Reilly and Kiyimba’s principles for rigor in qualitative research (transparency, integrity, transferability, reflexivity, ethicality), which we address throughout.^34^ Theoretically, our study is informed by Cranford’s conceptual framework of tensions between security and flexibility in home care.^35^

Cranford’s work emphasizes the complexities of home care dynamics, suggesting that there are tensions between workers’ quest for security and care recipients’ struggle for flexibility. These competing goals manifest for workers at the intimate level through gendered oppressions, “racialized indignities,” and other forms of relational and emotional harms that many care workers endure daily.^35(p5)^ For Cranford, security at the labor market level includes unionized environments, job security, and benefits like sick pay. Security-flexibility tensions are mediated by the local context, with some programs delivering high flexibility for care recipients but low security for workers, while other programs achieve a semblance of balance.^35^

### Study Setting

DF is one home care option among a variety of home and community care programs in Canada. Canada does not have a national home care system, with most programs delivered provincially.^36^ In most cases, including in the study sites, DF home care is used by a small proportion of home care clients.^37^ We selected two major cities in two provincial sites—Manitoba and Alberta. The research took place between June 2021 and April 2022. The sites were selected based on the team’s prior research that identified active DF programs in these provinces and program eligibility that allows care workers to be hired directly or through a third-party agency.^37,38^ Of note, Alberta was piloting a program for DF clients who want to hire agencies. Ethics approvals were obtained from [redacted] and participants gave written or verbal informed consent.

### Participant Recruitment and Selection

Workers were recruited through social media ads, snowball sampling, and community organizations. Participants were eligible if they worked currently, or had worked previously, in DF home care settings in Manitoba (Self and Family-Managed Care program) or Alberta (Self-Managed Care Program or Invoicing Program). Due to the difficulty of recruiting care workers, we did not employ any exclusion criteria. We prioritized the inclusion of women and migrant workers, and aimed for a diverse sample overall (eg, race/ethnicity, gender, age, birth country, education level, income), as informed by Cranford’s framework and our commitment to intersectional representation.^39-41^ Thematic saturation tends to occur around 12 participants given enough shared characteristics and we oversampled to account for diversity.^42^ Twenty workers participated in our study, and no participants withdrew.

### Data Collection

The first author, Kelly, conducted remote semi-structured in-depth interviews with 20 workers by phone or video. The interviews were roughly half an hour to an hour long and were audio-recorded. Our use of semi-structured, in-depth interviews contributes to the transparency of the research, including related concepts of trustworthiness and credibility by providing nuanced accounts of participants’ lived experiences and allowing “thick descriptions.”^34^ Transcription was manually completed by a third-party company.

Interview guides were developed by team members in consultation with a community advisory board. The care workers were asked about their career trajectories and working conditions. Open-ended questions were designed to elicit stories and opinions such as “What made you get into this line of work?”, “What do you think are the advantages and disadvantages of working directly for a client versus working for an agency” and “Do you intend to work in frontline care for a long time, or do you have other career plans?” Questions also prompted participants to reflect on their experiences, such as “How do you manage conflict with a client or their family?” and “What advice would you give a person considering entering this line of work?” A small honorarium or gift card was offered to all participants who completed an interview. Pseudonyms are used in all publications.

### Analysis

Authors Kelly, Dansereau, and Sebring performed axial coding and thematic analysis using Dedoose software (Table 1). Each transcript was coded by at least two of the authors to increase rigor, trustworthiness, and credibility as well as to check our assumptions (reflexivity). We used Dedoose’s embedded memo feature to track analytical insights as they emerged and reviewed the coding process and theme development at weekly team meetings to ensure transparency and reflexivity. Any disagreements or differences in interpretation were discussed among authors until resolved. Cranford’s framework directly informed the development of themes by allowing us to consider participants’ experiences as they related to the intimate level and labor market level as well as relevant personal (eg, age, work experience) and material factors (eg, pay, working conditions) that significantly shaped worker career trajectories and experiences.^35^ In developing our themes, we also paid attention to disconfirming cases as to ensure transparency.^34^

**Table 1.** Coding Scheme.

Theme	Codes	Description
Intimate level: Workers value flexibility, autonomy and respect found in direct hire relations	Relationship with family	Describing good rapport and/or tensions with family and other unpaid caregivers
	Career trajectory	Mentions of other jobs or phases in career
Labor market level: Agencies provide job security and supervisory supports with lower wages	Conflict and abuse (Worker POV)	Mentions of navigating conflict or experiencing abuse
	Long term plans and security	Quotations about if workers intend to stay in home care for a long time and/or if they feel their job is secure
	Agency	How workers characterize working for an agency
Labor market level: Care work as a steppingstone for immigration and citizenship	Career trajectories	Mentions of other jobs or phases in career
	Training	Quotations about any school or training the participant has done
	Culture/ethnicity/immigration	References of one’s culture, ethnicity, as it relates to care work, or experiences of immigration

## Results

### Participants

Fifteen of our 20 participants currently worked directly for their clients, however most also had experience in other settings, including non-profit and for-profit care agencies, retirement residences, nursing homes, and hospitals. The gender breakdown of our participants reflects care worker demographics more broadly in that 16 participants identified as women while 4 identified as men. A large proportion of participants had worked in care for more than 10 years. All but 3 had at least some post-secondary level training, and half had a bachelor’s degree or higher. For a summary of participant demographics, see Table 2.

**Table 2.** Participant Characteristics.

Self-identified gender	Woman	16
	Man	4
	Other gender	0
Age *One missing	25-44	10
	45-64	9
Born in Canada	Yes	12
	No	8
Highest level of education *One missing	High school	4
	Post-secondary	6
	Bachelor’s degree	5
	Graduate degree	4
Financial wellbeing	Sometimes/often have trouble	8
	Never have trouble	9
	Prefer not to answer	3
DF employer type	Direct hire	15
	Agency	5
Canadian work experience *(years)*	Less than 2 years	2
	2-10 years	10
	More than 10 years	8

## Findings

Through exploring work experiences and choice of work setting, we identify a range of personal and material factors at the intimate and labor market level that workers weigh when choosing their work setting, and specifically whether to work directly for a client or through an agency.

## Intimate Level: Workers Value Flexibility, Autonomy, and Respect in Direct Hire Relations

Participants in our study often began their careers working for home care agencies or in institutional settings. From there, they were sometimes offered a position by a former client. This theme highlights how workers tended to enjoy greater flexibility and autonomy in DF as compared to other care arrangements and were often able to develop trusting and mutually respectful relationships between themselves, their employers, and their clients.

### Flexibility

Our participants indicated that the flexibility allowed to DF clients through arranging their care services also resulted in more flexibility for workers. Flexibility for workers in this context refers to clients and families respecting the personhood of workers, for example, by allowing negotiations over scheduling, being open to discussing when and how care tasks would proceed, and listening to the advice and concerns of their workers.

An example of such flexibility and respect is provided by Debby. Debby is a middle-aged, Canadian-born woman who is a highly experienced care worker and holds a certificate in recreation and has worked predominantly in institutional settings. At the time of the interview, she had stepped back from full-time work and retained only one part-time position in direct hire. Debby emphasized the flexibility and support she was given by her employers, the adult children of an older woman with advanced dementia who was challenging to care for.

Debby: *I actually had quit, put in my notice over a year ago due to how bad the behaviour was getting towards me . . . the family they had said OK what do you want to do? Do you want to only work X number of days and we’ll try and find somebody else for those other days? . . .*

*And then I came back and said well, I’ll stay on with the knowledge that if she’s miserable and I feel like I need to take that whole—you know the catch phrase—mental health break . . . then that’s what I’ll have to do. And they said oh perfect! Ever since then there’s been times where they’re going, “Debby you know we’ve been reading in the notes”—because we write out notes every day—“what’s going on. She’s been getting a little miserable. Do you need to take a day off?” And some days it’s like yeah, you know what? That might be a good idea. And then other days it’s no I’m OK.*

Debby indicated she could only remain working with the difficult client precisely because of her employer’s support and flexibility which allowed her to care for her mental health.

For most participants, the flexibility of working directly for a client was an important advantage over working for agencies or in a facility. However, some participants indicated that flexibility could be a double-edged sword, particularly for those who are not strong negotiators, may be hesitant to speak up, younger or relatively inexperienced, or felt that the clients’ needs outweigh their own preferences.

Dave, a highly experienced worker with disability support certificate, indicated that his extensive knowledge of high-needs clients allowed him to navigate challenging situations that others may not be able to handle. Dave is a younger Canadian-born man who had previously managed multiple staff and clients at a disability support agency but left the “*very demanding role*” because “*it wasn’t paying the bills*.” He then found a full-time office job but remained in care work through a weekend-position for 1 client. In the excerpt below Dave muses as to why someone may prefer to have the back-up of an agency.

Dave: *He can’t breathe on his own. So, like there’s a lot of danger and touchiness around that. Sometimes his ventilator will fail, and you have to breathe manually for him with a pump . . . And so because there’s all of that crazy touchy stuff in the air his wife can get short. And I know she has with other people.*

*For myself . . . I know my rights as a worker and all of that. I don’t feel like I would be in a situation I couldn’t handle. But I could see someone being very worried about where they would go if they had a problem or a conflict with the person that they were working for.*

When workers were able to develop high quality relationships at the intimate level, it tended to allow more flexibility among all parties. Some relationships blurred into being treated “like family,” and participants predominantly perceived such relations positively.

### Autonomy and Respect

Workers reported feeling higher levels of autonomy and respect in working directly with a client than in other care positions. Respect meant being treated with humanity, including having their ideas, concerns and suggestions listened to. Autonomy meant having the capacity to make decisions about how care unfolded, and being entrusted to do so.

Raquel’s experience exemplifies how working directly for a client can provide more autonomy than an agency. Raquel (age unknown), an immigrant woman from west Africa, had earned a master’s degree in social work in her home country and moved to Canada just over two years prior to her interview. Although her social work credentials were accepted by the provincial college, she had not been able to secure a job in her profession and planned to return to university to earn Canadian credentials. She was working full time in a group home run by a small agency and had a part time DF job to “*help pay the bills*.” In the quote below, Raquel compares the two positions, and describes how having to go through a manager or supervisor can add delays or communication problems; for Raquel, talking directly with families “*makes the job easier*.”

Raquel: *Sometimes [with the agency] you can’t reach your manager. . . and things aren’t attended to as quick as you’d like. Working with the family we have direct access . . . you can make suggestions and because the family knows that you work one-on-one with that person . . . They take you seriously and they just work better with you to get things done.*

Raquel went on to emphasize that being respected and heard was highly important to her:

Raquel: *The family that I work for, even if I get a high-paying job tomorrow, and I had an opportunity to keep part-time hours with them I would still do that. It’s not about the money. It’s about the way I’m treated.*

Dave also felt more respected in the intimate dynamics of working directly for client, commenting how “*thoughtful*” the family he worked for was, and commending them as understanding both the “*worker role*” and “*the way things should be*.”

### Labor Market Level: Agencies Provide Job Security and Supervisory Supports With Lower Wages

Working for agencies or other care organizations provide some job security, supportive supervision, conflict mediation, and generally standardized hours. Workers in agencies can also “pick up” extra shifts and more readily change clients or take on new clients. Security and support come at the cost of reduced flexibility at the intimate level, and notably lower wages.

### Job Security and Supervisory Support

Raquel, previously quoted, preferred working directly but nevertheless suggested why someone might prefer working for an agency:

Raquel: *. . . For the agency there’s a lot of structure. Your hours, inside your hours there’s vacant shifts that you can pick up if you’re not working full-time, but [working] for the family it’s not really guaranteed. Plus, if your client dies that’s the end of your job.*

Workers who preferred agencies appreciated having a supervisor who could act as an intermediary in difficult situations such as unreasonable expectations, inappropriate sexual advances, or racist and derogatory treatment.

Anne, a highly experienced, middle-aged immigrant woman with a health care aid certificate, was working part time for a client and part time for an agency. She spoke about conflict and difficulties with various clients and described how an agency provided her with support in such situations:

Anne : *I like working for the agency [. . .] you have a clear guide to follow. And then if it’s something that is not in your care guide, you—the person asks you to do, you can politely say, “I’m sorry. I know exactly what to do, what I’m not supposed to be doing.” If they—some of them can start to be very verbally [abusive], I will leave . . . You call the agency and you leave. [. . .]*

*So if it was working for a private person, how are we supposed to fix that? I have no choice, I could just quit. I have a choice with the agency, I say “I do not want to go there anymore.” I say, “Do not send me there” and that was it.*

Brooke’s story also provides examples of situations that may be prevented through the supervision of an agency. Brooke, a Canadian-born woman in her 20s with no formal training or certification, landed her first formal care job in a full-time overnight position working directly for her client. She described the position as poorly paid at $17 per hour, with long shifts and no overtime pay.

Brooke: *The fact that you’re not really a trained person, and his life is in your hands. I had to bag him a few times to help him breathe, because he was having problems with his trach. There was some stuff that I probably shouldn’t have been doing that I was doing.*

*. . . it ended up being a sexual harassment type of situation . . . he would ask me to do things to his body that were not protocol, I guess you’d call it. But I thought it was. Because obviously, he knew that I didn’t know procedure. I did what was asked of me, and it was it was terrible. And it escalated. I definitely wasn’t the only victim of this man. I’m just the only person that said anything about it.*

*I would definitely go through a company now, through an actual agency. Unionised, whatever the case may be. Because there’s just way too much room for error and for people to be taken advantage of.*

Her inexperience and lack of training put her at risk of exploitation across multiple dimensions, ranging from low pay and long hours to sexual harassment. Brooke quit the position just prior to her interview and was planning on going back to school to pursue a career in counselling or mental health.

Unfair manipulation may alternatively occur when there is a strong emotional connection between the client and worker. This exploitation may be difficult to avoid as workers may feel relational pressures to respond to needs in a “family-like” manner. Jeff, an older, Canadian born man approaching retirement age, held a health care aid certificate and had worked in disability, home care and other care contexts since the 1980s and then moved out of care work following a back injury in the early 2000s. A decade later he was asked to come back by a previous client and, at the time of his interview, was working full time for this client. Jeff felt that their long-standing relationship put him under some degree of moral obligation to be constantly available regardless of his own health and wellbeing.

Jeff: *I think the thing that is the toughest when you work with self-managed is that—and it depends on the personality but—where you almost become a part of the family and there’s almost like a dependency that kind of gets built into it. He’s always said, “If you’re sick I still want you to come in because you know how hard it is for me to find somebody to fill in for you.”*

Working directly for a client in DF involves more precarious and uncertain work conditions than other care work. Workers in this context face the possibility of entering an abusive situation and having to manage on their own because they lack alternative supports.

Workers with more experience and training were better equipped to negotiate and handle difficult situations, as vocalized by Danielle, a younger immigrant woman, who holds a nursing degree from southeast Asia and has worked in care for more than a decade: *“Knowledge, like knowledge is really a power for workers or else you’re going to get abused.”*

### Low Wages

At the time of her interview, Danielle was working full-time in a private facility for people with dementia, and part-time working directly for a family in DF. She was paid slightly more per hour in DF than at her full-time facility job, but the DF position lacked benefits such as dental insurance. Both of her current jobs paid more than her previous position with a private home care agency:

Danielle: *Privately it’s $57 for two hours. [At the supportive living residence. . .] a little less. When I worked before with the agency, the agency is just paying me $16, which is very low. [The] family treated me really nice, and they told me the ins and outs on how much they pay. So for example they’re paying the agency $32, I’m getting $16.*

The details of pay vary significantly across our participants: Danielle, as noted above, reported high pay in direct hire at $28.50 per hour, however other direct-hire participants reported pay in the range of $19 to $25. Most participants worked for agencies as a second and part-time job, and there was consensus across all participants that agencies pay the least (starting at minimum wage—$11.95/h in MB at the time of our study, and $15/h in AB) and rarely provide benefits.

## Labor Market Level: Care Work as a Steppingstone for Immigration and Citizenship

Broadly, non-professional care work, whether in home care or in a facility, served as a stepping stone to immigration and citizenship for participants in our study. Yet, all but one had a foreign-earned professional degree. Their foreign credentials overwhelming went unrecognized, and some sacrificed considerable professional prestige to earn a living in Canada.

Recently immigrated participants typically transitioned into working directly with clients after first working in other care settings. The following quotes illustrate their motivations regarding their education, and the career trajectory within Canada that led them to their position.

Isa, a younger woman, earned a nursing degree in southeast Asia specifically to support her goal of emigrating.

Isa: *I was in my 20s, 22 when I graduated nursing. I was influenced by my parents. Yes, I was young back then and then they said, oh, go to nursing, it will take you somewhere else. Yeah, and it took us here [to Canada].*

Danielle, quoted in the previous theme, similarly earned a nursing degree with the goal of emigrating from southeast Asia. She moved to Canada in 2008 through an employer-sponsored visa with a private care facility where she was still working at the time of the interview. Once established with a permanent residency she added a second job working for a home care agency, eventually moving to working with a DF family “*as they can pay more*.”

Steven, also from southeast Asia, was a registered nurse specialist. He moved to Canada through the live-in caregiver program where the family acts as an employer-sponsor. His move was difficult as he was not receiving a livable wage; his original employer agreed to release him so he could look for a different opportunity. He was hired by a different family who agreed to sponsor him as a live-in caregiver with his wages paid through DF:

Steven: *. . . If I am to weigh in how much I was earning back home in [southeast Asia] compared to how much I’m just earning here in Canada [with the first family], it just doesn’t add up. If I had known that this would be the only pay, I would have just stayed back home. . . . [but] I’m already here . . . so I decided to just look for a new employer*

*I was really desperate . . . I was telling them that I badly needed this job because that’s my—I have no money, nothing. I can’t thank them enough for the opportunity and their graciousness, [but] it’s really a step back for me. Because I really have to swallow my ego. You have your pride. I’m an RN, not only that, but [nursing speciality] RN*

Steven’s contract was to last for another two years, during which time he planned to earn Canadian-recognized nursing credentials.

Working in care in Canada often meant a significant loss of wages or professional prestige for newly immigrated participants, yet it was a trade-off that many felt was worth it. Working directly for clients rarely launched their career paths; rather, they tended to find their first Canadian jobs in other non-professional care settings, particularly facility-based positions, that were perhaps more straightforward to find. Once established in Canada, they tended to move into DF for similar reasons as the Canadian-born participants; that is, better wages, and more flexibility and autonomy than other forms of care work.

## Discussion

Our study set out to understand the experiences and factors that home care workers consider when choosing a work setting, and specifically what influences their decisions to work directly for a client. Drawing on Cranford’s work, we found people weigh and balance personal and material trade-offs at the intimate and labor market level when choosing their setting of work.

The results highlight the relevance of flexibility at the intimate level, where workers *and* clients get input into the conditions of work/care which then improves the degree of “intimate security”^35^ for both. Most care work is task-and-time based. Due to the complexity and variety of tasks completed by home care workers, it is “difficult to measure the time required to do work *well.*”^43,44^ Truly transforming home care past a task-and-time framework would require considering a salary-based rather than shift-based model of employment. This would enable workers and clients the flexibility required of this dynamic.

Flexibility can work *for* the worker and *against* the worker. For example, many workers in our study found the flexibility of their schedule allowed them to attend to other responsibilities outside of their job when needed, and this may be a benefit for those looking to get into DF work.^45^ In other cases, this flexibility led to blurred boundaries between the responsibilities of the client’s family and that of the worker^9^ as noted by participant Jeff. These situations, what Cranford refers to as “the economy of favours”^35^ may be partially mediated in the home setting by working for an agency, however this is unclear. This is not to say that close relationships *must* be avoided, as close and trusting relationships are a requirement for high quality care.^46^

Workers also value increased autonomy and respectful relationships in working directly for a client. Existing research documents that workers value the autonomy they experience in home care work,^26-28^ and our study suggests even further levels of autonomy in direct hire situations. It is important to recognize autonomy as a valuable perk of the job—one that is often interpreted as a risk. For example, Cranford’s framework warns, “It is important not to overemphasize the importance of security at the intimate level over labor market security . . . the way the work is set up . . . shapes the relationship between workers and recipients” (p. 39). The workers in our study highlight working directly comes with a valuable degree of autonomy and satisfaction. Of course, it is important not to diminish the real risks of these arrangements, as many workers have limited options for addressing workplace issues. In some jurisdictions there are non-profit, third-party organizations that have been proposed or are in operation (eg, in Alberta, ERAPs^47^; proposed in Ontario, see Dansereau et al^48^ that offer a mechanism to support workers and mediate some of the risk born by workers who wants to work in settings with high degrees of autonomy.

At the labor market level, home care agencies and institutional care settings provide enhanced security with the major trade-off of lower wages. This finding adds to the care literature that documents differences in pay among care settings; such differences are not just between long-term care facilities and home care,^29^ but even among types of home care employment arrangements.^24,32^ In a system straining to recruit enough workers, consistency in pay may be a key factor for the transition toward more home-based services.^24,49^

The study reinforces findings that care work is a stepping stone for recent immigrants attempting to gain citizenship. Institutional or agency employment arrangements rather than direct employment through DF are seen as more “legitimate” work experiences.^5,19^ Among our participants working in an institutional setting as an entry point to the workforce, many were also taking on second jobs in home care. Many also expressed a preference for home-based work but were unable to move out of their facility positions due to the reality of lower pay and lack of benefits in home care positions. Given Canada’s current shortage of health care workers,^50^ policymakers need to reconsider full employment of these recent immigrants, given their professional designations in their home countries. To illustrate, only 35% of foreign-trained nursing graduates are working in the nursing profession in Canada compared to 90% of Canadian-trained.^51^

Further, the participant experiences in this study suggest that care worker wages should be standardized across institutional, home care and other work settings to support health system transformation toward more home and community-based services. Care workers should be provided opportunities for autonomous practice and the first-hand knowledge generated through their work experience should be recognized and fostered in all care work settings. This may include greater recognition of international training and experience to account for the wealth of specialized, professional knowledge that many immigrant care workers bring with them from health professional careers in foreign countries. In Canada, for example, the federal government recently launched the Foreign Credential Recognition Program to address such concerns.^52^

### Limitations

This study was limited to two Canadian provinces using directly-funded home care and the findings must be considered in light of these specific contexts. However, the findings may be transferable to other programs or care settings in different geographic contexts given that our participants had experience working in a variety of care settings. At times, we have limited reporting on the specificities of participants’ social locations (eg, gender, race), education, work experience, or other personal characteristics to protect their anonymity, however, this also limits the degree to which we can comment on the relevance of these factors across our dataset.

## Conclusion

It is critically important to listen to the narratives of frontline care workers describing the trade-offs and decision-making process as they find work. Care workers themselves offer solutions for how they can be supported to work in their preferred setting, with high degrees of autonomy and fair and consistent compensation.
