# Corrigendum to “Wages or Legitimacy? A Qualitative Analysis of Home Care Worker Perspectives in Choosing Work Settings”

**DOI:** 10.1177/00469580241277426

**Published:** 2024-08-22

Kelly C, Dansereau L, Sebring JCH, Lee Y, Williams A. Wages or Legitimacy? A Qualitative Analysis of Home Care Worker Perspectives in Choosing Work Settings. INQUIRY: The Journal of Health Care Organization, Provision, and Financing. 2024;61. doi:10.1177/00469580241248094

In the above-referenced article funding section has been updated and correct funding section reads as:

The author(s) disclosed receipt of the following financial support for the research, authorship, and/or publication of this article: This research was supported by funding from the Canadian Institutes of Health Research (CIHR), Institute of Health Services and Policy Research (PJT-169001). This research was supported by the Chung-Ang University Research Grants in 2022.

